# Optimizing training sets to identify superior genotypes in hybrid populations

**DOI:** 10.3389/fpls.2025.1699491

**Published:** 2026-01-15

**Authors:** Szu-Ping Chen, Chen-Tuo Liao

**Affiliations:** Department of Agronomy, National Taiwan University, Taipei, Taiwan

**Keywords:** genomic best linear unbiased prediction model, genomic prediction, hybrid performance, plant breeding, whole genome regression model

## Abstract

The identification of superior hybrids from candidate populations is a central goal in plant breeding, particularly for commercial applications and large-scale cultivation. In this study, several promising training set optimization methods in genomic selection (GS) are evaluated and extended to construct predictive models for the identification of top-performing genotypes in hybrid populations. The methods investigated include: (i) a ridge regression-based approach, ${\text{MSPE}}_{\left(\text{v}2\right)}^{\text{Ridge}}$, (ii) a generalized coefficient of determination-based method, ${\text{CD}}_{\text{mean}(\text{v}2)}$, and (iii) an A-optimality-like ranking strategy, ${\text{GV}}_{\text{average}}$. To assess predictive performance in identifying genotypes with the highest true breeding values (TBVs), three evaluation metrics were developed. Since TBVs are latent quantities derived from models, simulation experiments based on real genotype data from wheat (*Triticum aestivum* L.), maize (*Zea mays*), and rice (*Oryza sativa* L.) were carried out to assess the proposed methods. Results demonstrated that ${\text{GV}}_{\text{average}}$ not only achieved substantial computational efficiency but also generally generated highly informative training sets across a broad range of sizes. However, when constructing small training sets, ${\text{GV}}_{\text{average}}$ occasionally failed to maintain adequate genomic diversity. In such cases, ${\text{CD}}_{\text{mean}(\text{v}2)}$ is recommended as a more reliable alternative. Overall, the proposed framework provides a flexible and effective approach to optimizing training sets for hybrid breeding, thereby enhancing the accuracy of genomic prediction in practical breeding programs.

## 1 Introduction

Genomic selection (GS) has become an indispensable strategy in modern plant breeding for the identification of superior genotypes from large breeding populations. The central principle of GS is the capture of quantitative trait loci across the genome using dense molecular markers (Meuwissen et al., 2001). Genomic prediction models are then constructed to estimate genomic estimated breeding values (GEBVs) for individuals in the breeding population based on a training set comprising both phenotype and genotype data. Since the accuracy of GS is highly dependent on the quality of phenotype data and the genomic relationship between the training set and the target population, optimization of the training set has become a critical factor for the successful implementation of GS (Wu et al., 2023).

Considerable research has focused on developing optimization methods for training sets, especially in inbred populations (Muleta et al., 2019; Sabadin et al., 2022; Fernandez-Gonzales et al., 2023). A wide variety of strategies—ranging from minimizing prediction error variance (PEV) to maximizing the generalized coefficient of determination (CD)—have been proposed (Rincent et al., 2012; Isidro et al., 2015; Rincent et al., 2017; Akdemir et al., 2015; Ou and Liao, 2019; Akdemir and Isidro-Sánchez, 2019). These methods, however, generally assume additive marker effects and have been designed primarily for inbred populations. A recent comprehensive review by Alemu et al. (2024) highlighted the development of these approaches and emphasized the need for more flexible methods applicable to a broader range of breeding scenarios.

In practice, plant breeders are often tasked not only with improving inbred lines but also with identifying superior hybrids for commercial deployment. Hybrid breeding exploits heterosis, or hybrid vigor, to enhance yield, stress tolerance, and quality traits in crops. The performance of a hybrid depends on both general combining ability (related to additive effects) and specific combining ability (linked to dominance and epistatic effects). Consequently, training set optimization for hybrid populations must explicitly account for nonadditive genetic components. Despite its practical importance, this area has received far less attention than inbred-focused optimization. For example, Guo et al. (2019) evaluated several data-mining strategies for representative subset selection in hybrid breeding and demonstrated their advantages over random sampling and earlier CD- or PEV-based methods.

More recently, Chen et al. (2024) proposed and validated training set optimization methods for inbred populations that consider only additive effects. Building on these advances, the present study extends such approaches to hybrid populations by integrating both additive and dominance marker effects into predictive modeling. The present study attempts to identify superior hybrids with the highest true breeding values (TBVs). As TBVs of hybrids are latent quantities derived from models, comprehensive simulation studies based on real genotype data from wheat (*Triticum aestivum* L.), maize (*Zea mays*), and rice (*Oryza sativa* L.), respectively, presented in Zhao et al. (2015); Technow et al. (2014), and Xu et al. (2018), were conducted to evaluate the proposed methods.

This work aims to (i) adapt and evaluate state-of-the-art training set optimization methods for hybrid breeding, (ii) compare their predictive ability and computational efficiency, and (iii) provide practical recommendations for method selection under different training set sizes. Our findings contribute to bridging the methodological gap between inbred- and hybrid-focused optimization and offer breeders flexible tools for enhancing genomic prediction in hybrid breeding programs.

## 2 Materials and methods

### 2.1 Genome datasets

Three genome datasets were used in this study and are described as follows.

#### 2.1.1 Wheat

Zhao et al. (2015) investigated the genome-based establishment of a high-yielding heterotic pattern for hybrid wheat breeding. Their study was based on 135 advanced elite winter wheat lines, which include 15 male lines and 120 female lines. A set of 1,604 hybrids derived from crosses between the male and female lines was evaluated for grain yield (GY, Mg/ha) across 11 environments. The adjusted means of the GY were available in the dataset. For the genotype data, the 135 parental lines were genotyped by using a 90,000 SNP array based on an Illumina Infinium array. After quality tests, 17,372 high-quality SNP markers were retained. In the current study, the genotype data with 1,604 hybrids and 17,372 SNPs were used for simulation experiments.

#### 2.1.2 Maize

Technow et al. (2014) analyzed a maize dataset comprising 123 dent lines and 86 flint lines, in which the genotype data consist of 35,478 SNP markers across the 209 lines. A total of 10,578 single-cross hybrids was obtained between the 123 dent lines and the 86 flint lines. Only 1,254 out of the 10,578 possible hybrids were measured on the two traits: GY (in quintals per hectare) and grain moisture content (GM, in percent). The genotype data with 1,254 hybrids and 35,478 SNPs were used for simulation experiments in our study.

#### 2.1.3 Rice

A hybrid rice population was presented in Xu et al. (2018), consisting of 575 hybrid combinations produced by crossing five male sterile lines with 115 inbred lines. The 120 parental lines were sequenced, and the SNPs were filtered with a missing rate > 0.2 in the male sterile lines and 0.5 in the 115 inbred lines, retaining 2,561,889 SNPs. The filtered SNPs aligned with the 3kRG core SNP set v.4 (a total of 996,009 SNPs), leaving 116,482 SNPs remaining. The aligned SNP markers were further filtered by missing rate ≥ 0.05 and minor allele frequency ≤ 0.1, resulting in 63,735 SNPs. Phenotype data, including eight traits, were evaluated at two locations. In our study, the genotype data with 575 hybrids and 63,735 SNPs were used for simulation experiments.

Details regarding the quality filtering criteria, imputation methods, and normalization procedures for the above datasets can be found in the cited references. To examine the relative effects of different training set sizes across the crop genome datasets, 500 hybrids were randomly sampled from each dataset to serve as the candidate population. These selected candidate populations were designated as WHEAT.CP, MAIZE.CP, and RICE.CP. Let $w_{ij}^{A}$ be the additive effect score for the locus of individual *i* at SNP *j* for $i=1,\;2,\;\cdots ,\;n_{c}$ and j=1, 2, ⋯, p, where $n_{c}$ is the number of individuals in the candidate population, and p is the number of SNP markers. From VanRaden (2008) and Wu et al. (2019), the additive effect scores were coded as:


$$w_{ij}^{A}=\{\begin{array}{c} 1,\;\text{if}\;AA\;\\ 0,\;\text{if}\;AB\\ -1,\;\text{if}\;BB, \end{array}$$

where *AA* denotes the homozygote of the major allele, *AB* denotes the heterozygote, and *BB* denotes the homozygote of the minor allele at locus *j*. Moreover, the corresponding dominance effect scores $w_{ij}^{D}$ were coded as:


$$w_{ij}^{D}=\{\begin{array}{c} 0,\;\text{if}\;AA\;\\ 1,\;\text{if}\;AB\\ 0,\;\text{if}\;BB. \end{array}$$

Normalized marker scores were then used in analysis throughout this study. Let $x_{ij}^{A}$ and $x_{ij}^{D}$ respectively denote the corresponding normalized marker scores for additive and dominance effects, then $x_{ij}^{A}=(w_{ij}^{A}-{\bar{w}}_{j}^{A})/s_{j}^{A}$ where ${\bar{w}}_{j}^{A}$ and $s_{j}^{A}$ are the sample mean and the sample standard deviation of all $w_{ij}^{A}$ values in SNP j. Likewise, $x_{ij}^{D}$ were obtained in the same manner. Furthermore, genomic relationship matrices for the additive and dominance effects were calculated as:

(1) $$K_{A}=\frac{X_{A}X_{A}^{T}}{p}$$

and

(2) $$K_{D}=\frac{X_{D}X_{D}^{T}}{p}$$

where $X_{A}$ and $X_{D}$ are the normalized marker score matrices for additive and dominance effects of the candidate population, respectively.

### 2.2 Training set optimization methods

The methods for training set optimization were derived mainly from two kinds of statistical models: whole genome regression (WGR) models and genomic best linear unbiased prediction (GBLUP) models.

#### 2.2.1 A whole-genome regression model-based method

The WGR model, including both additive and dominance effects, is expressed as:

(3) $$y=\beta_{0}1_{n}+X_{A}\beta_{A}+X_{D}\beta_{D}+e,$$

where y represents the vector of phenotypic values; $\beta_{0}$ denotes the constant term; $1_{n}$ is the unit vector of length *n* (with *n* being the number of phenotypic values); $\beta_{A}$ corresponds to the vector of additive effects; $\beta_{D}$ corresponds to the vector of dominance effects; and e denotes the vector of random errors.

The model described in Equation 3 can be rewritten as:


$$y=\beta_{0}1_{n}+X\beta +e,$$

where ***X*** = [***X****_A_*, ***X****_D_*] and $\beta ={\left(\beta_{A},\beta_{D}\right)}^{T}$. Following Chen et al. (2024), a mean square of prediction error (MSPE) method has been proposed for evaluating the entire candidate population:

(4) $${\text{MSPE}}^{\text{Ridge}}=1+\frac{1}{n_{c}}\{Tr[X_{\text{c}}AA^{⊤}X_{\text{c}}^{⊤}]+Tr[(X_{\text{c}}-X_{\text{c}}AX_{t}){\left(X_{\text{c}}-X_{\text{c}}AX_{t}\right)}^{T}]\},$$

Where $X_{\text{c}}$ and $X_{t}$ are defined as the merged marker score matrices of additive and dominance effects for the candidate population and the training set, respectively, and


$$A=X_{t}^{⊤}{\left(X_{t}X_{t}^{⊤}+\lambda I_{n_{t}}\right)}^{-1}.$$

Here, $n_{t}$ is the training set size and λ is the regularization parameter. Note that the curly brackets in Equation 4 were missing in the original formulae presented by Chen et al. (2024); the correct formulae can be found in Appendix B of their paper. Minimizing ${\text{MSPE}}^{\text{Ridge}}$ in Equation 4 is equivalent to minimizing the following simplified version:

(5) $${\text{MSPE}}_{\left(\text{v}2\right)}^{\text{Ridge}}=Tr[X_{\text{c}}AA^{⊤}X_{\text{c}}^{⊤}]+Tr[(X_{\text{c}}-X_{\text{c}}AX_{t}){\left(X_{\text{c}}-X_{\text{c}}AX_{t}\right)}^{T}]$$

The marker scores were replaced by principal components (PCs) scores to reduce computing time, and the first PCs were selected such that the cumulative explained variance exceeded 99%. Moreover, the regulation parameter λ was fixed at 1 when calculating ${\text{MSPE}}_{\left(\text{v}2\right)}^{\text{Ridge}}$ in the current study.

#### 2.2.2 A genomic best linear unbiased prediction model-based method

A GBLUP model considering both additive and dominance effects was used to construct training sets, which can be described as:

(6) $$y=1_{n}\mu +{ɡ}_{A}+{ɡ}_{D}+e,$$

where μ is the general mean; ${ɡ}_{A}$ and ${ɡ}_{D}$ are respectively the vectors of genotypic values for the additive and dominance effects, and e is the vector of random errors. It is assumed that ${ɡ}_{A},$ ${ɡ}_{D},$ and e are mutually independent and distributed with multivariate normal distributions, denoted by ${ɡ}_{A}\sim N(0,\;\sigma_{A}^{2}K_{A}),$ ${ɡ}_{D}\sim N(0,\;\sigma_{D}^{2}K_{D}),$ and $e\sim N(0,\;\sigma_{e}^{2}I_{n}),$ where $\sigma_{A}^{2}$ and $\sigma_{D}^{2}$ are respectively the genetic variances for additive and dominance effects, $K_{A}$ and $K_{D}$ are the corresponding genomic relationship matrices, and $\sigma_{e}^{2}$ is the error variance.

The GBLUP model described in Equation 6 can be rewritten as:

(7) $$y=1_{n}\mu +g+e,$$

where $ɡ={ɡ}_{A}+{ɡ}_{D}$ and $ɡ∼N(0,\;\sigma_{A}^{2}K_{A}+\sigma_{D}^{2}K_{D})$. Following Chen et al. (2024), a heuristic-based CD method for evaluating the entire candidate population can be described as follows. Let ${ɡ}_{t}$ be the true gnomic values for a selected training set as described in Equation 7. The BLUP for ${ɡ}_{t}$ is given by:


$${\hat{ɡ}}_{t}={\left(M_{t}+G_{t}^{-1}\right)}^{-1}M_{t}y_{t}$$

where $M_{t}=I_{n_{t}}-{\bar{J}}_{n_{t}}$, $G_{t}=\alpha_{A}K_{At}+\alpha_{D}K_{Dt}$, and $y_{t}$ consists of the phenotypic values in the training set. Here, ${\bar{J}}_{n_{t}}$ is the square matrix of order $n_{t}$ with all elements equal to $1/n_{t}$, and $\alpha_{A}=\sigma_{A}^{2}/\sigma_{e}^{2},$ $\alpha_{D}=\sigma_{D}^{2}/\sigma_{e}^{2},$ $K_{At}$ and $K_{Dt}$ are the additive and dominance relationship matrices for the training set. Moreover, let $g_{c}$ be the true genomic values in the candidate population, then the BLUP for $g_{c}$ is given by:


$${\hat{g}}_{c}=G_{ct}{\left(M_{t}G_{t}+I_{n_{t}}\right)}^{-1}M_{t}y_{t}$$

where $G_{ct}=\alpha_{A}K_{Act}+\alpha_{D}K_{Dct}$. Here, $K_{Act}$ and $K_{Dct}$ are the additive and dominance relationship matrices between the candidate population and the training set. Furthermore, the variance–covariance matrix for ${\hat{g}}_{c}$ is equal to the covariance matrix between $g_{c}$ and ${\hat{g}}_{c}$, which is:

(8) $$Var({\hat{g}}_{c})=Cov(g_{c},{\hat{g}}_{c})=(G_{ct}{\left(M_{t}G_{t}+I_{n_{t}}\right)}^{-1}M_{t}G_{ct}^{T})\sigma_{e}^{2}$$

Let $A_{i}$ denote the ith diagonal element of $G_{ct}{\left(M_{t}G_{t}+I_{n_{t}}\right)}^{-1}M_{t}G_{ct}^{T}$ in Equation 8, and let $B_{i}$ denote the corresponding element in $G_{c}=\alpha_{A}K_{Ac}+\alpha_{D}K_{Dc}$, for $i=1,\;2,\;\ldots ,\;n_{c}$, where $K_{Ac}$ and $K_{Dc}$ separately denote the additive and dominance relationship matrices for the candidate population. A heuristic-based CD criterion is given by:

(9) $${\text{CD}}_{\text{mean}(\text{v}2)}=\sum_{i=1}^{n_{c}}\frac{{\left[cov(g_{ci},{\hat{g}}_{ci})\right]}^{2}}{var(g_{ci})\times var({\hat{g}}_{ci})}=\sum_{i=1}^{n_{c}}(\frac{{\left[A_{i}\right]}^{2}}{B_{i}\times A_{i}})=\sum_{i=1}^{n_{c}}(\frac{A_{i}}{B_{i}}).$$

Maximizing ${\text{CD}}_{\text{mean}(\text{v}2)}$ is equivalent to maximizing the mean of squared correlations between the true and estimated genotypic values in the candidate population. The regulation parameters of $\alpha_{A}$ and $\alpha_{D}$ were both arbitrarily fixed at 1 when calculating ${\text{CD}}_{\text{mean}(\text{v}2)}$, because the selection of training sets based on the CD criterion is usually not affected by the setting of the parameter values (Rio et al., 2022; Fernandez-Gonzales et al., 2023). We will further discuss the robustness of this setting. An exchanging algorithm implemented in the R package TSDFGS (Ou, 2022) was utilized to optimize training sets for individually minimizing ${\text{MSPE}}_{\left(\text{v}2\right)}^{\text{Ridge}}$ in Equation 5 or maximizing ${\text{CD}}_{\text{mean}(\text{v}2)}$ in Equation 9.

#### 2.2.3 A-optimality-like method

The A-optimality criterion is used to minimize the average variance for estimating model parameters in the field of classical optimal designs. Conversely, the ${\text{GV}}_{\text{average}}$ criteria here was utilized to construct training sets that maximize the genomic variation. This is because the probability of correctly identifying the true superior genotypes from a candidate population can be enhanced if the training set can explain the variation of genotypic values as much as possible (Tanaka and Iwata, 2018; Tsai et al., 2021; Tu and Liao, 2024).

For a fixed training set size $n_{t}$, Chen et al. (2024) proposed an A-optimality-like method to construct training sets such that the average variance for the selected $g_{t}$ is maximized. The method, modified for the GBLUP model in Equation 7, can be described as:


$${\text{GV}}_{\text{average}}=arg_{d^{*}\in S_{d}}\;max(Tr[K_{d}]),$$

where $S_{d}$ denotes the set comprising all possible subsets with $n_{t}$ candidates, and $K_{d}$ is the submatrix of $\sigma_{A}^{2}K_{Ac}+\sigma_{D}^{2}K_{Dc}$ corresponding to d. The variance components $\sigma_{A}^{2}$ and $\sigma_{D}^{2}$ were both fixed at 1 when calculating ${\text{GV}}_{\text{average}}$. The ${\text{GV}}_{\text{average}}$ method ranks all candidates based on their individually calculated metrics and selects the top-ranked ones, eliminating the need for computationally intensive algorithms.

### 2.3 Evaluation metrics

Let ${\hat{v}}_{\left(1\right)}$, ${\hat{v}}_{\left(2\right)}$, …, ${\hat{v}}_{\left(n_{c}\right)}$ be the GEBVs corresponding to the TBVs $v_{\left(1\right)}\geq v_{\left(2\right)}\geq \ldots \geq v_{\left(n_{c}\right)}$ in a candidate population. By reordering these GEBVs, it follows that ${\hat{v}}_{\left(\pi_{1}\right)}\geq {\hat{v}}_{\left(\pi_{2}\right)}\ldots \geq {\hat{v}}_{\left(\pi_{n_{c}}\right)},$ where $\pi =(\pi_{1},\pi_{2},\ldots ,\pi_{n_{c}})$ is a permutation of $\pi_{0}=(1,\;2,\ldots ,\;n_{c}).$ Notably, $\pi_{1},$ $\pi_{2}$, …, $\pi_{n_{c}}$ are actually the ranks of TBVs corresponding to ${\hat{v}}_{\left(\pi_{1}\right)},$ ${\hat{v}}_{\left(\pi_{2}\right)},$ $\ldots ,\;\;{\hat{v}}_{\left(\pi_{n_{c}}\right)}.$ Three evaluation metrics given below were used to assess the predictive performance of a training set in identifying the best k genotypes with the highest TBVs in the candidate population.

#### 2.3.1 Normalized discounted cumulative gain

Blondel et al. (2015) proposed the normalized discounted cumulative gain (NDCG) score at position *k*, defined as follows. The discounted cumulative gain (DCG) at position *k* of the predicted ranking $\pi =(\pi_{1},\pi_{2},\ldots ,\pi_{n_{c}})$ and the ideal ranking $\pi_{0}=(1,\;2,\ldots ,\;n_{c})$ based on a training set are as follows:

(10) $$DCG@k(v,\pi (\hat{v}))=\sum_{i=1}^{k}f(v_{\left(\pi_{i}\right)})d(i),$$

(11) $$DCG@k(v,\pi_{0}(v))=\sum_{i=1}^{k}f(v_{\left(i\right)})d(i),$$

where f(v) is a monotonically increasing gain function and d(i) is a monotonically decreasing discount function. In this study, a linear gain function f(v)=v, and a discount function $d(i)=\frac{1}{{\text{log}}_{2}(i+1)}$ were used in Equations 10, 11. The NDCG score for identifying the best *k* genotypes is then defined as:

(12) $$NDCG@k(v,\hat{v})=\frac{DCG@k(v,\pi (\hat{v}))}{DCG@k(v,\pi_{0}(v))}$$

The $NDCG@k(v,\hat{v})$ value ranges from 0 to 1, with higher values indicating better performance of the training set.

#### 2.3.2 Spearman’s rank correlation

Spearman’s rank correlation (SRC) was applied to measure the linear relationship between the ranks of the top *k* genotypes with the highest GEBVs and their true ranks in TBVs. It is defined as:


$$SRC@k=\frac{\sum_{i=1}^{k}(i-\frac{k+1}{2})(\pi_{i}-\bar{\pi })}{\sqrt{\left[\sum_{i=1}^{k}{\left(i-\frac{k+1}{2}\right)}^{2}]\times [\sum_{i=1}^{k}{\left(\pi_{i}-\bar{\pi }\right)}^{2}\right]}},$$

where $\bar{\pi }=\sum_{i=1}^{k}\pi_{i}/k.$ The SRC@k actually is the Pearson’s correlation calculated from the paired values of $\left(i,\;\pi_{i}\right)$ for i=1, 2, ⋯, k.

#### 2.3.3 Rank sum ratio

The sum of ranks in TBVs corresponding to the top *k* genotypes with the highest GEBVs is $\sum_{i=1}^{k}\pi_{i}$, and the sum of ranks of the ideal ranking is $\sum_{i=1}^{k}i$. The ratio of these two rank sums indicates the ability of the training set to identify the best *k* genotypes and is defined as:


$$RS\_ratio@k=\frac{\sum_{i=1}^{k}i}{\sum_{i=1}^{k}\pi_{i}}.$$

The $RS{\_}_{ratio}@k$ also ranges from 0 to 1, taking the value of 1 if the top *k* genotypes with the highest GEBVs exactly match the *k* genotypes with the highest TBVs.

### 2.4 Simulation studies

Based on the GBLUP model in Equation 6, phenotype data were simulated to evaluate the performance of the construction methods, including ${\text{MSPE}}_{\left(\text{v}2\right)}^{\text{Ridge}}$, ${\text{CD}}_{\text{mean}(\text{v}2)}$, and ${\text{GV}}_{\text{average}}$. Random sampling was also performed as a baseline method. For each candidate population (WHEAT.CP, MAIZE.CP, and RICE.CP), the additive and dominance relationship matrices $K_{A}$ and $K_{D}$ were first using Equations 1, 2. Subsequently, training sets were generated from each construction method at sizes of 50, 100, 150, and 200.

From some historical studies on crops such as wheat, rice, and maize (Hassan et al., 2013; Chen et al., 2023; Barreto et al., 2024; Al-Daej, 2023), 31 estimates of $\text{γ}=\sigma_{D}^{2}/\sigma_{A}^{2}$ were available for various traits. The mean and standard deviation of the 31 estimated γ values were 1.43 and 2.56, respectively. Therefore, the model parameters were fixed at μ=100, $\;\sigma_{A}^{2}=\;20,$ $\;\sigma_{D}^{2}=\text{γ}\times \;\sigma_{A}^{2}$ with γ=0.5, 1, 2,and 4 and $\sigma_{e}^{2}=\frac{1-h^{2}}{h^{2}}\times (\sigma_{A}^{2}+\sigma_{D}^{2})$ with $h^{2}=(\sigma_{A}^{2}+\sigma_{D}^{2})/(\sigma_{A}^{2}+\sigma_{D}^{2}+\sigma_{e}^{2})=0.3$ and 0.6 in this simulation study. Accordingly, 2,000 datasets were generated for each parameter setting, i.e., $g_{A}∼MVN(0,\sigma_{A}^{2}K_{A}),\;g_{D}∼MVN(0,\sigma_{D}^{2}K_{D}),$ and $e∼MVN(0,\sigma_{e}^{2}I_{n}).\;$ Simulated TBVs were obtained as the fixed general mean μ plus the simulated $g=g_{A}+\;g_{D},$ and simulated phenotypic values y were obtained as the simulated TBVs plus the residual e. The mean values and the standard deviations of NDCG@k, SRC@k, and $RS{\_}_{ratio}@k$ with k=20 across the 2,000 simulated datasets were calculated to compare the methods. Moreover, the relative improvement percentages (RIPs) across different metrics and optimization methods, calculated based on the random sampling method, were also reported. The RIP can be expressed as:

(13) $$\text{RIP}=\frac{{\bar{M}}_{i}-{\bar{M}}_{0}}{{\bar{M}}_{0}}\times 100$$

Here, ${\bar{M}}_{i}$ represents the mean value of a given metric under a specific optimization method, and ${\bar{M}}_{0}$ denotes the corresponding mean value obtained from the random sampling method. The Bayesian reproducing kernel Hilbert space (RKHS) method in the R package BGLR (Pérez and de los Campos, 2014) was used to perform the GEBV prediction.

## 3 Results

**Figure 1:**
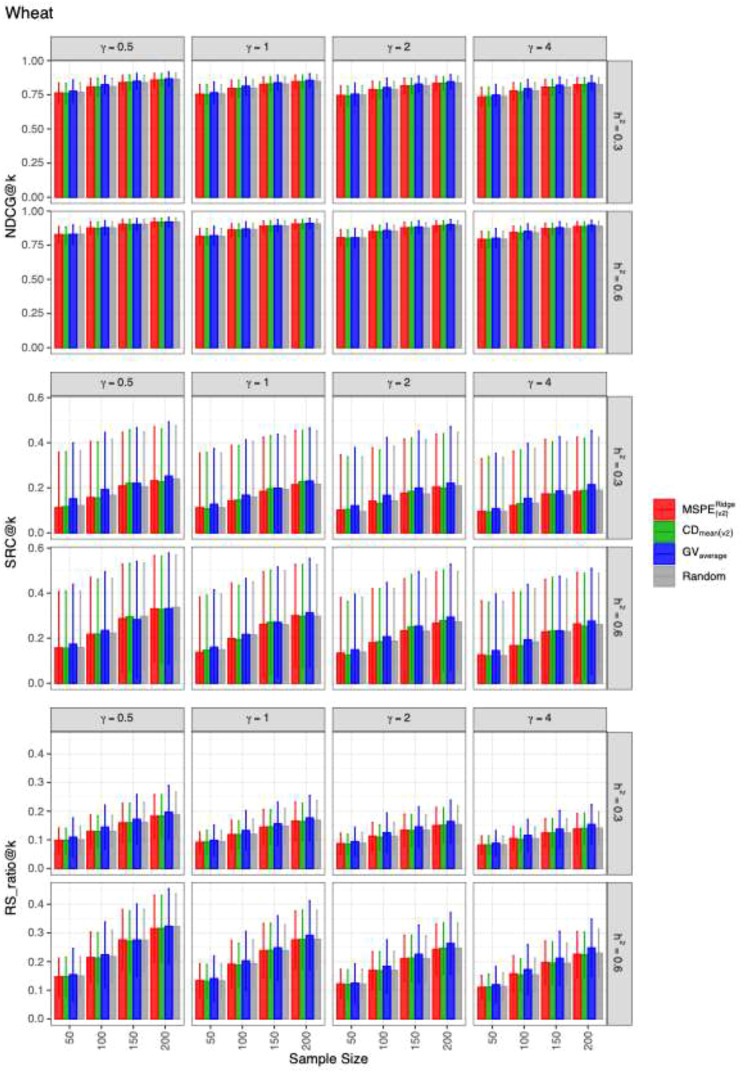
The means and the standard deviations of the resulting values of evaluation metrics for identifying the best 20 genotypes over 2,000 simulated datasets based on the training set construction methods in the WHEAT.CP dataset.

**Figure 2:**
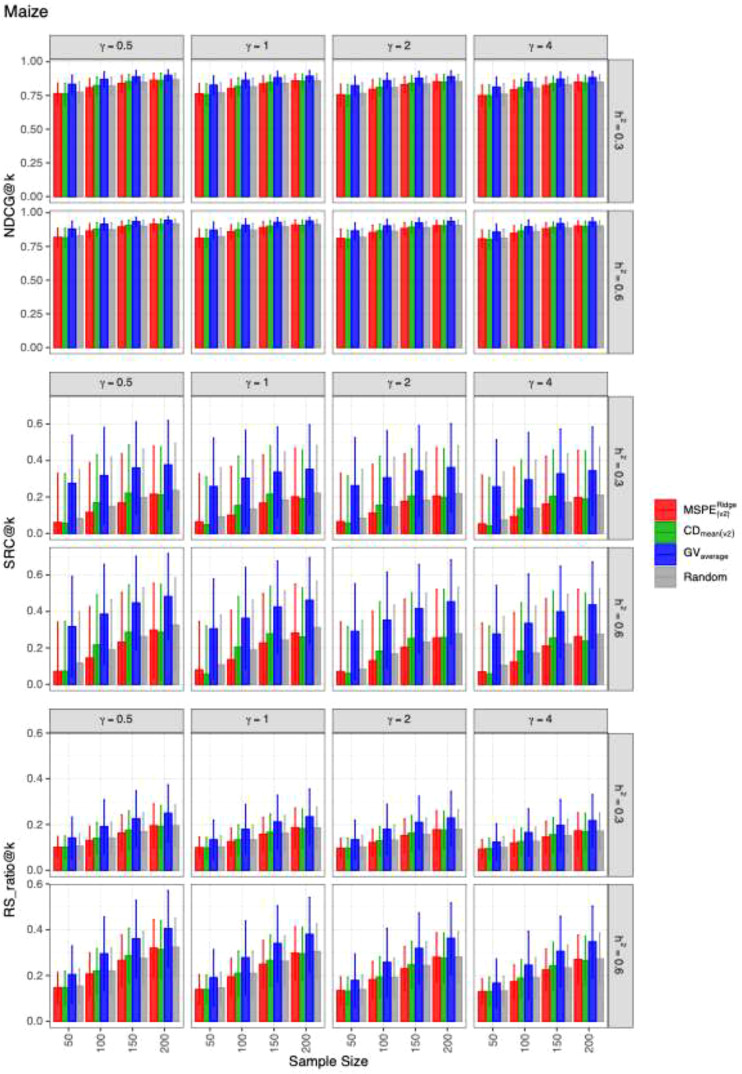
The means and the standard deviations of the resulting values of evaluation metrics for identifying the best 20 genotypes over 2,000 simulated datasets based on the training set construction methods in the MAIZE.CP dataset.

**Figure 3:**
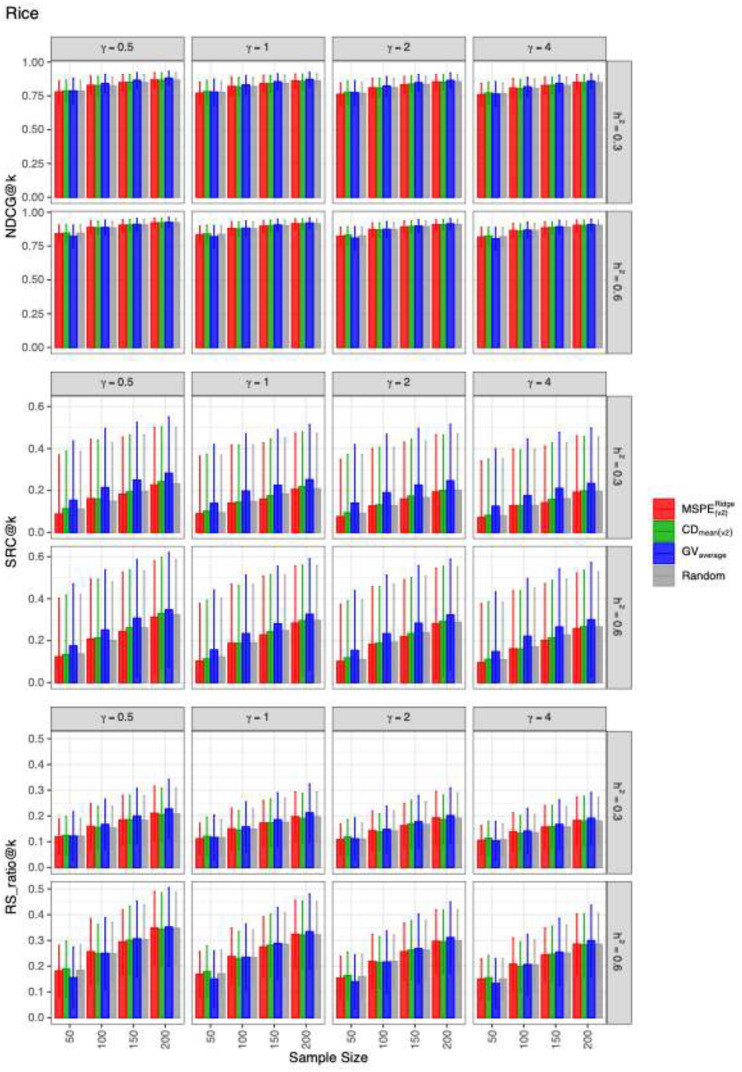
The means and the standard deviations of the resulting values of evaluation metrics for identifying the best 20 genotypes over 2000 simulated datasets based on the training set construction methods in the RICE.CP dataset.

The mean values and the standard deviations of the evaluation metrics for identifying the top 20 genotypes across 2,000 simulation runs for each dataset are presented in **Figures 1**–**3**. The RIPs, as defined in Equation 13, for all datasets are provided in **Supplementary Tables S1–S9**. In addition, the minimum and maximum RIPs across all simulation scenarios for each combination of evaluation metric and training set construction method are summarized in **Tables 1**–**3**. The key findings are as follows:

**Table 1:** The minimum and maximum relative improvement percentages across all simulation scenarios for the WHEAT.CP dataset.

Metrics		Methods		
		${\text{MSPE}}_{\left(\text{v}2\right)}^{\text{Ridge}}$	CD_mean(v2)_	GV_average_
NDCG@k=20	min	-0.507	-0.714	-0.131
	max	0.259	0.195	1.858
SRC@k=20	min	-8.954	-9.339	-4.668
	max	7.846	8.811	28.934
RS_ratio@k=20	min	-2.899	-3.001	0.305
	max	2.395	1.138	11.774

**Table 2:** The minimum and maximum relative improvement percentages across all simulation scenarios for the MAIZE.CP dataset.

Metrics		Methods		
		${\text{MSPE}}_{\left(\text{v}2\right)}^{\text{Ridge}}$	CD_mean(v2)_	GV_average_
NDCG@k=20	min	-1.747	-2.074	2.855
	max	-0.047	0.764	7.602
SRC@k=20	min	-33.902	-48.005	47.992
	max	-2.773	19.030	250.533
RS_ratio@k=20	min	-9.367	-5.642	24.263
	max	0.428	4.227	35.407

**Table 3:** The minimum and maximum relative improvement percentages across all simulation scenarios for the RICE.CP dataset.

Metrics		Methods		
		${\text{MSPE}}_{\left(\text{v}2\right)}^{\text{Ridge}}$	CD_mean(v2)_	GV_average_
NDCG@k=20	min	-0.861	-0.504	-2.869
	max	0.520	1.117	2.102
SRC@k=20	min	-21.196	-5.943	7.125
	max	8.564	8.558	55.926
RS_ratio@k=20	min	-3.881	-2.982	-15.337
	max	3.555	7.374	9.312

- Performance of construction methods: Overall, ${\text{GV}}_{\text{average}}$ generally outperformed the other three methods. Among them, ${\text{CD}}_{\text{mean}(\text{v}2)}$ performed slightly better than ${\text{MSPE}}_{\left(\text{v}2\right)}^{\text{Ridge}}$ and random sampling across most datasets and evaluation metrics. An exception occurred in the RICE.CP dataset, where ${\text{GV}}_{\text{average}}$​ underperformed the other methods at $n_{t}=50$ when estimating NDCG@k and RS_ratio@k in RICE.CP (**Figure 3**).
- Random sampling as a baseline: The random sampling method consistently exhibited competitive performance relative to ${\text{MSPE}}_{\left(\text{v}2\right)}^{\text{Ridge}}$ and ${\text{CD}}_{\text{mean}(\text{v}2)},$, suggesting that the heuristic-based optimization methods do not always confer large advantages.
- Dataset-specific differences: Method performance varied across datasets. In the MAIZE.CP dataset, ${\text{GV}}_{\text{average}}$ substantially outperformed the other three methods across all evaluation metrics. By contrast, in WHEAT.CP and RICE.CP, the four methods showed more similar levels of performance.
- Effect of dominance-to-additive variance ratio ( γ): For fixed $n_{t}$ and $h^{2},$, the predictive ability of all methods gradually declined as γ​ increased, indicating that higher relative dominance variance reduced accuracy.
- Effect of training set size ( $n_{t}$): For fixed γ and $h^{2},$, the performance of all methods improved with increasing training set size, consistent with expectations from GS theory.
- Effect of heritability ( $h^{2}$): For fixed γ and $n_{t}$, all methods benefited from higher heritability, highlighting the importance of genetic signal strength in prediction accuracy.
- Metric-specific comparisons: Across datasets and scenarios, the differences among construction methods were generally smaller for *NDCG@k* compared with *SRC@k* and RS_ratio@k, suggesting that *NDCG@k* may be less sensitive to differences in training set optimization.

## 4 Discussion

The simulation studies demonstrated that ${\text{GV}}_{\text{average}}$ generally outperformed ${\text{MSPE}}_{\left(\text{v}2\right)}^{\text{Ridge}},$ ${\text{CD}}_{\text{mean}(\text{v}2)},$, and random sampling methods across the datasets and training set sizes, except when the training set size was small ( $n_{t}=50$) in the RICE.CP dataset. Note that the minimal RIPs of metrics in RICE.CP for ${\text{GV}}_{\text{average}}$ occurred in cases with $n_{t}=50$. This decline in performance likely reflects reduced diversity in the training set. ${\text{GV}}_{\text{average}}$ prioritizes candidates with the highest genomic variation, which may inadvertently select individuals with high relatedness, thereby limiting the genetic diversity of the training set. For example, if two candidates with the highest genomic variation are nearly identical, ${\text{GV}}_{\text{average}}$ would likely select both, thereby reducing the overall genomic diversity of the training set. In contrast, heuristic-based approaches use exchange algorithms to maintain diversity, which is especially beneficial for small training sets.

**Figure 4:**
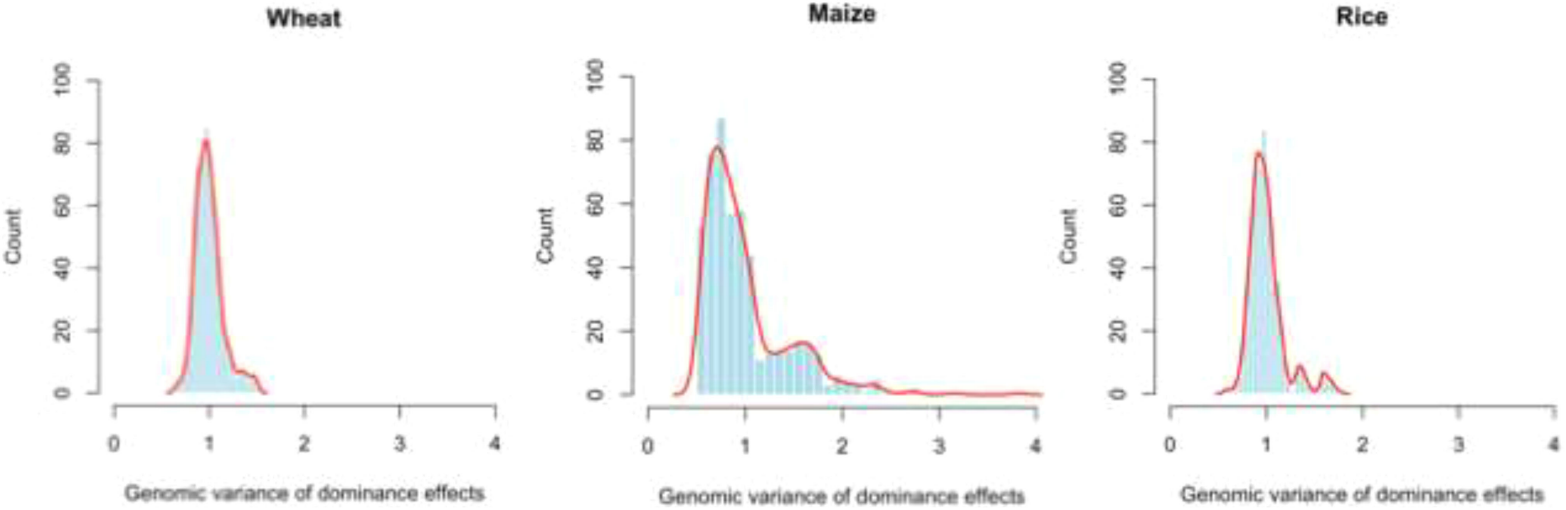
Histograms of genomic variances attributable to dominance effects for genotypes in the WHEAT.CP, MAIZE.CP, and RICE.CP datasets.

The ${\text{MSPE}}_{\left(\text{v}2\right)}^{\text{Ridge}}$ and ${\text{CD}}_{\text{mean}(\text{v}2)}$ methods for training set selection were primarily designed to maximize the relationship between the training set and the candidate population. In contrast, the ${\text{GV}}_{\text{average}}$ method focuses on maximizing the variation of dominance effects, thereby expanding the search space for identifying superior hybrids. Notably, ${\text{GV}}_{\text{average}}$ exhibited a clear advantage in the maize dataset, likely due to maize displaying more pronounced dominance effects than wheat and rice. The genomic variance attributable to dominance effects for genotypes in the WHEAT.CP, MAIZE.CP, and RICE.CP datasets is displayed in **Figure 4**, indicating that maize exhibited substantially stronger dominance effects than wheat and rice. This finding helps explain why ${\text{GV}}_{\text{average}}$ showed a marked advantage over the other methods in the MAIZE.CP dataset. Moreover, small training sets can hinder accurate estimation of dominance relationships among hybrids, which may also explain the weaker performance in small training set scenarios.

Another consistent observation from the results was that differences among construction methods were smaller for *NDCG@k* than for *SRC@k* and RS_ratio@k. This observation can be attributed to the fact that *NDCG@k* in Equation 12 incorporates both the simulated TBV and a discount function. In our simulations, the simulated TBV was defined as the sum of the general mean and the simulated genotypic value, with the general mean set substantially larger than the genotypic value. As a result, the metric became less sensitive to differences in DCG values between the predicted and ideal rankings. This result is also be supported by the fact that the estimates for *NDCG@k* had much smaller standard deviations than those for *SRC@k* and RS_ratio@k (**Figures 1**-**3**).

To check whether the methods of ${\text{MSPE}}_{\left(\text{v}2\right)}^{\text{Ridge}}$ and ${\text{CD}}_{\text{mean}(\text{v}2)}$ are affected by the parameter setting when constructing training sets, 20 random training sets each comprising 50 genotypes were chosen from the MAIZE.CP dataset, which has a higher dominance effect compared to the WHEAT.CP and RICE.CP datasets. The ${\text{MSPE}}_{\left(\text{v}2\right)}^{\text{Ridge}}$ values in Equation 5 were calculated at λ= 0.01, 0.1, 1, 10, and 100, and the ${\text{CD}}_{\text{mean}(\text{v}2)}$ values in Equation 9 were calculated with $(\alpha_{A},$ $\alpha_{D})$ equal to (0.5, 0.5), (0.5, 1), (1, 1), (1, 2), and (1, 4) for the 20 training sets. These results are displayed in **Supplementary Tables S10, S11**. For ${\text{MSPE}}_{\left(\text{v}2\right)}^{\text{Ridge}}$, the results in **Supplementary Table S10** showed that rankings of candidate training sets remained identical, indicating robustness to the choice of λ. From **Supplementary Table S11**, Pearson’s correlation and Spearman’s rank correlation between the resulting ${\text{CD}}_{\text{mean}(\text{v}2)}$ values at $(\alpha_{A},$ $\alpha_{D})=(1,\;1)$ (default setting) and the other settings are displayed in **Table 4**. Both of the correlation coefficients decreased as the discrepancy of the dominance variance component increased. However, the correlation analyses across different weight settings of additive and dominance components still demonstrated strong concordance with the default setting, supporting its use in practice. To check the robustness for ${\text{GV}}_{\text{average}}$, $\sigma_{A}^{2}K_{Ac}+\sigma_{D}^{2}K_{Dc}$ was calculated with $\left(\sigma_{A}^{2},\sigma_{D}^{2}\right)$ equal to (0.5, 0.5), (0.5, 1), (1, 1), (1, 2), and (1, 4). The overlap proportion of the top 50 candidates and RS_ratio@k=50 between the default setting of $\left(\sigma_{A}^{2},\sigma_{D}^{2})=(1,1\right)$ and the other settings are displayed in **Table 5**. These results showed high stability under alternative parameter settings of variance components, further reinforcing its practical utility with default parameters. The robustness verification based solely on these rank concordance tests may be insufficient. A more comprehensive sensitivity analysis—considering a wider range of parameter values, runtime comparisons, and changes in evaluation metrics—would provide stronger evidence. We plan to address this issue in future work.

**Table 4:** Pearson’s correlations (*r*) and Spearman’s rank correlations (SRC) over the CD_mean(v2)_ values of the 20 random training sets between the default setting, ( $\alpha_{A},\alpha_{D}$) = (1,1), and the other four settings in the MAIZE.CP dataset.

( $\alpha_{A},\alpha_{D}$)	(0.5,0.5)	(0.5,1)	(1,2)	(1,4)
*r*	0.9816	0.9962	0.9926	0.9712
SRC	0.9880	0.9910	0.9820	0.9444

**Table 5:** The overlap proportion of the top 50 candidates and RS_ratio@k=50 between the default setting, ( $\sigma_{A}^{2},\sigma_{D}^{2}$) = (1,1), and the other four settings for the ${\text{GV}}_{\text{average}}$ method in the MAIZE.CP dataset.

$\left(\sigma_{A}^{2},\sigma_{D}^{2}\right)$	(0.5,0.5)	(0.5,1)	(1,2)	(1,4)
Overlap proportion	50/50	50/50	50/50	50/50
RS_ratio@k=50	1.0000	1.0000	1.0000	1.0000

We employed the randomly selected datasets of WHEAT.CP, MAIZE.CP, and RICE.CP throughout our study. To evaluate the potential effect of stochastic sampling of candidate populations, we conducted additional simulations at $n_{t}=50$ across 50 randomly sampled candidate populations per dataset. These simulation results are displayed in **Supplementary Figures S1–S3**, which were consistent with those obtained from a single candidate population (**Figures 1**-**3**), confirming the robustness of our conclusions. Notably, ${\text{GV}}_{\text{average}}$ continued to underperform in the small training sets for the rice dataset, particularly in estimating NDCG@k and RS_ratio@k at $h^{2}=0.6$ (**Supplementary Figure S3**), an outcome consistent with earlier findings (**Figure 3**).

The ranking agreement metrics of *NDCG@k*, *SRC@k*, and *RS_ratio@k* may have limited biological interpretability, as they are not directly linked to actual genetic gain. Nevertheless, because the present study focuses on identifying the truly superior genotypes within a candidate population, these metrics remain valuable for quantifying information gain from the selected training sets. There is, however, still room for developing evaluation metrics with stronger biological relevance. Furthermore, the simulation framework in this study was primarily based on the GBLUP model described in Equation 6, which incorporates most factors that may influence the identification of superior genotypes in hybrid populations. Despite this, the model still lacks some degree of practical and biological realism. Future studies could improve upon this by relaxing the assumptions of normality and independence of genotypic values derived from additive and dominance effects. Alternatively, empirical estimates of model parameters from real datasets could be employed to enhance biological validity.

The simulation studies conducted for performance comparison evaluated the effects of the training sets within a single breeding cycle. However, it is well recognized that other key factors, such as genetic variation, can substantially influence genetic gain over multiple breeding cycles (Lehermeier et al., 2017; Chung and Liao, 2022). Future research could therefore explore the long-term impact of training sets constructed using the proposed methods. Moreover, the phenotypic value of a trait is influenced by genotype, environment, and genotype-by-environment interactions. In practice, local environmental conditions can markedly affect hybrid performance during the growth period. Consequently, superior hybrids identified through simulation may not necessarily perform as expected under real field conditions. Thus, conducting extensive field experiments across different crops and environments to validate the main findings of this study would be highly valuable.

The ranking method based on ${\text{GV}}_{\text{average}}$ is expected to be substantially more computationally efficient than the heuristic-based approaches of ${\text{MSPE}}_{\left(\text{v}2\right)}^{\text{Ridge}}$ and ${\text{CD}}_{\text{mean}(\text{v}2)}$. Nevertheless, concerns may still arise regarding the actual computation time. Accordingly, we reported in **Table 6** the runtimes required to generate training sets of sizes 50, 100, 150, and 200 for the WHEAT.CP, MAIZE.CP, and RICE.CP datasets. The runtimes ranged from 745.08 to 6,602.93 min, from 148.18 to 1,359.55 min, and from 0.001 to 0.0276 s employing ${\text{MSPE}}_{\left(\text{v}2\right)}^{\text{Ridge}}$, ${\text{CD}}_{\text{mean}(\text{v}2)}$, and ${\text{GV}}_{\text{average}}$ respectively. It should be noted that computational time may escalate with an increase in training set size or candidate population size. The time requirement, however, can be mitigated by employing upgraded hardware and optimized software implementations.

**Table 6:** Runtime of executing R code for the construction methods to generate training sets in the datasets.

Dataset	*n*_t_	${\text{MSPE}}_{\left(\text{v}2\right)}^{\text{Ridge}}$	${\text{CD}}_{\text{mean}(\text{v}2)}$	${\text{GV}}_{\text{average}}$
WHEAT.CP	50	1,016.93 min	148.47 min	0.0276 s
	100	1,658.43 min	350.65 min	0.0276 s
	150	2,526.76 min	749.50 min	0.0276 s
	200	6,602.93 min	1,343.47 min	0.0276 s
MAIZE.CP	50	798.43 min	148.18 min	0.0015 s
	100	1,286.43 min	349.07 min	0.0015 s
	150	1,959.26 min	757.95 min	0.0015 s
	200	2,822.26 min	1,357.42 min	0.0015 s
RICE.CP	50	745.08 min	148.67 min	0.0010 s
	100	1,216.91min	394.67 min	0.0010 s
	150	1,790.75 min	756.07 min	0.0010 s
	200	2,588.75 min	1,359.55 min	0.0010 s

Note that the programs were run on a PC server with a 3.49-GHz, 16-core AMD Ryzen 9 3950X.

Overall, these results reinforce the value of ${\text{GV}}_{\text{average}}$ as a computationally efficient and generally competitive method for training set optimization in hybrid populations. While Chen et al. (2024) showed its competitiveness in inbred populations, the present study extends its utility to hybrids by incorporating dominance effects into the construction framework. ${\text{GV}}_{\text{average}}$ can thus be recommended for medium-to-large training sets where efficiency and scalability are critical. However, for small training sets, ${\text{CD}}_{\text{mean}(\text{v}2)}$ remains preferable due to its ability to safeguard diversity and maintain predictive accuracy. Finally, the practical contribution of this study is underscored by the availability of an R code implementing the proposed methods (https://github.com/spcspin/AD), providing breeders with accessible tools for optimizing training sets in hybrid breeding programs.

## 5 Conclusion

This study provides a comprehensive evaluation of training set optimization methods for GS in hybrid populations by explicitly incorporating both additive and dominance effects. Our findings demonstrate that ${\text{GV}}_{\text{average}}$ offers a fast and effective strategy for constructing training sets of medium to large sizes, while ${\text{CD}}_{\text{mean}(\text{v}2)}$ is more reliable in small-sample scenarios, where maintaining genetic diversity is critical. These insights not only extend existing optimization frameworks from inbred to hybrid breeding but also provide practical recommendations for method selection under varying breeding program conditions. Future research could explore integrating epistatic effects, multienvironment data, and dynamic training set updating strategies to further enhance the accuracy and robustness of genomic prediction in hybrid breeding.

## Data Availability

WHEAT.CP, MAIZE.CP, and RICE.CP datasets and the 20 training sets of size 50 sampled from the MAIZE.CP dataset for the robustness verification can be downloaded from the GitHub repository (https://github.com/spcspin/AD).
