# Supplementary material for: Optimizing training sets to identify superior genotypes in hybrid populations
Source: Front Plant Sci. 2026 Jan 15;16:1699491. doi: 10.3389/fpls.2025.1699491 (PMC12854142; doi:10.3389/fpls.2025.1699491)
Supplement: Supplementary file 1 [file DataSheet1.pdf]

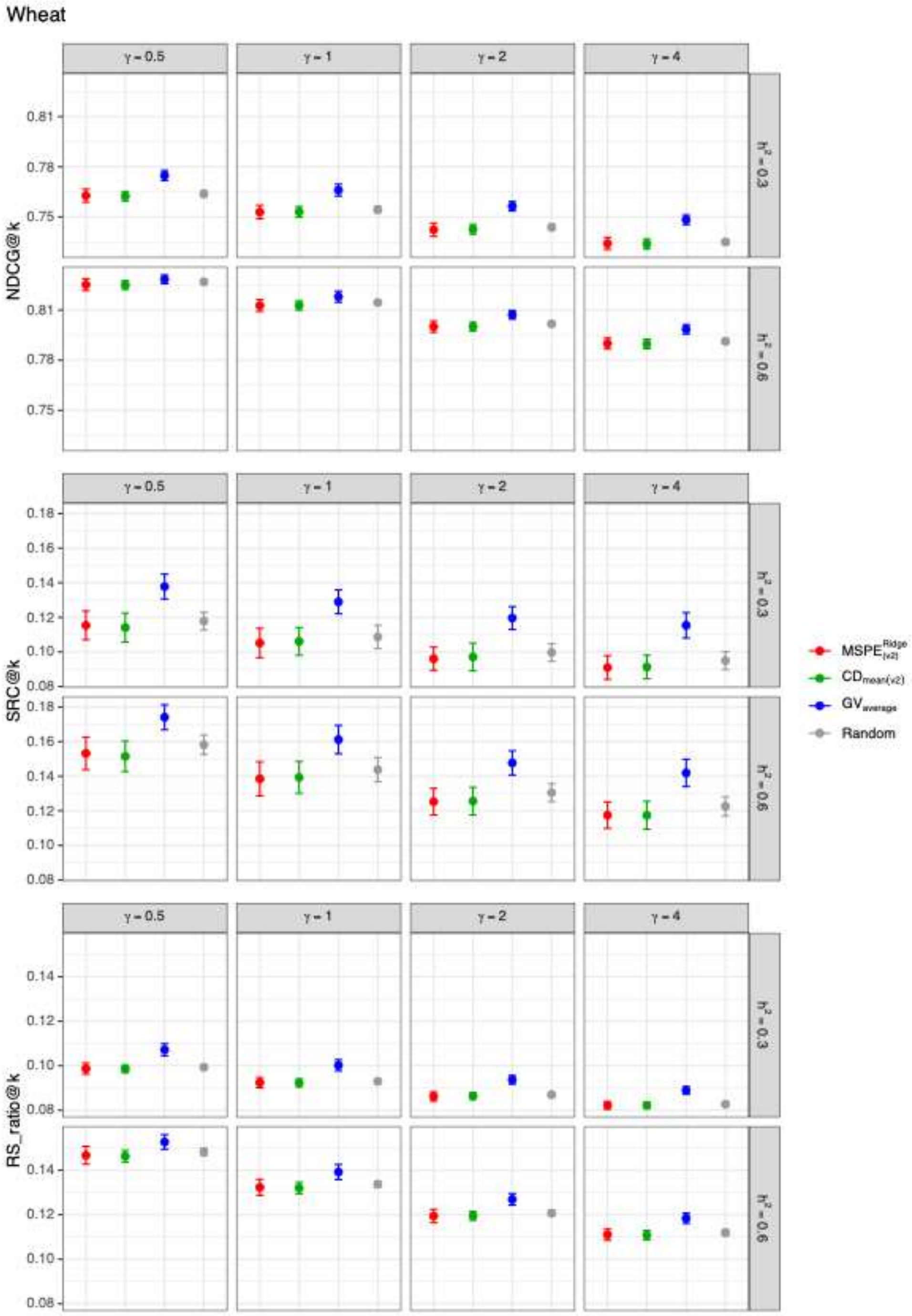

Figure S1. The mean ± sd (standard deviation) plots of average evaluation metrics for identifying the best 20 genotypes over 50 candidate populations based on the training sets with size $n_t = 50$ constructed from the methods in the wheat dataset

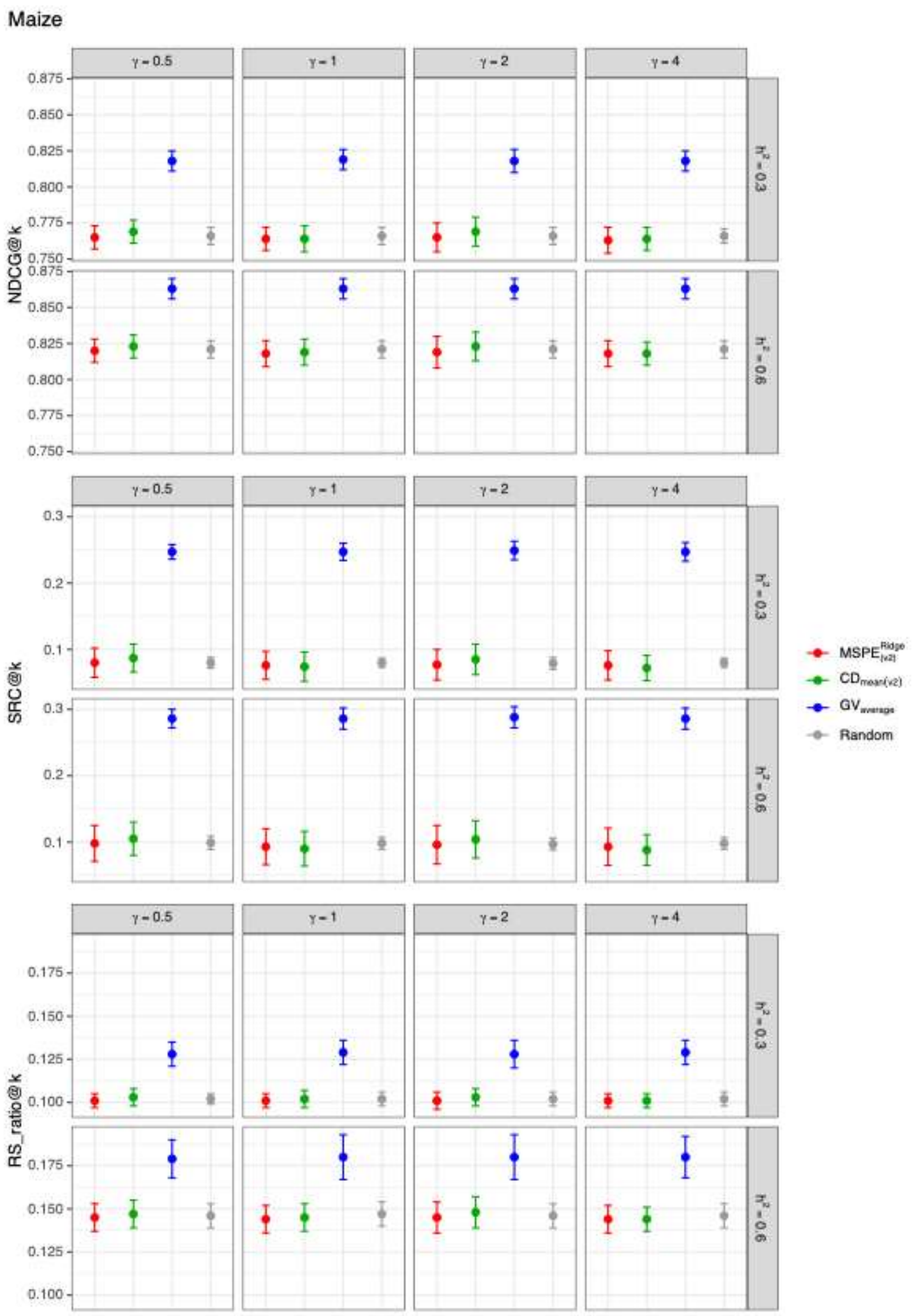

Figure S2. The mean ± sd (standard deviation) plots of average evaluation metrics for identifying the best 20 genotypes over 50 candidate populations based on the training sets with size $n_t = 50$ constructed from the methods in the maize dataset

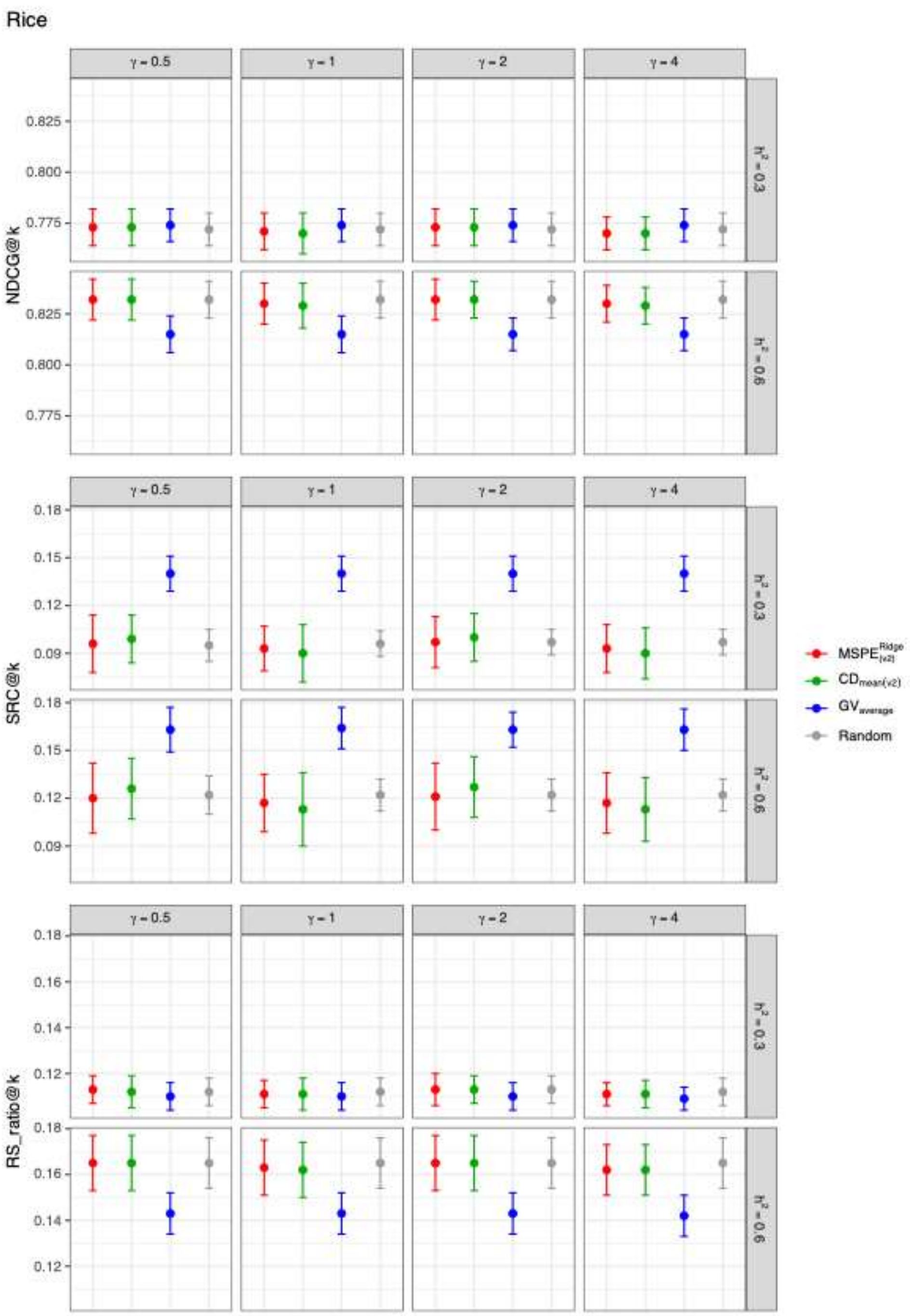

Figure S3. The mean $\pm$ sd (standard deviation) plots of average evaluation metrics for identifying the best 20 genotypes over 50 candidate populations based on the training sets with size $n_t = 50$ constructed from the methods in the rice dataset

Table S1. The relative improvement percentage of $NDCG@k = 20$ in the WHEAT.CP dataset

γ	h^2	n_t	$MSPE_{(v2)}^{Ridge}$	$CD_{mean(v2)}$	$GV_{average}$
0.5	0.3	50	-0.5075	-0.7147	1.2056
		100	-0.2316	-0.2890	1.7768
		150	-0.0053	0.1955	1.1910
		200	-0.3946	-0.3192	0.6531
	0.6	50	-0.3224	-0.4681	-0.1319
		100	-0.1054	-0.4230	0.1748
		150	0.0419	-0.0812	-0.0875
		200	-0.2129	-0.1603	0.0433
1	0.3	50	-0.3059	-0.5865	1.1891
		100	-0.2568	-0.2422	1.8078
		150	-0.2211	0.1261	1.1787
		200	-0.2502	-0.2537	0.8799
	0.6	50	0.0607	-0.1851	0.7753
		100	-0.2862	-0.5116	0.5060
		150	-0.0912	0.0095	0.3816
		200	-0.1213	-0.0452	0.4610
2	0.3	50	-0.3004	-0.7066	1.1028
		100	-0.0027	-0.4422	1.8583
		150	-0.0479	0.0081	1.5308
		200	-0.4681	-0.2310	1.1276
	0.6	50	0.2597	-0.2458	0.1451
		100	-0.0585	-0.1622	0.8405
		150	0.0431	0.1871	0.7824
		200	-0.2734	-0.0353	0.6746
4	0.3	50	-0.4100	-0.2485	1.4346
		100	-0.0612	-0.7367	2.0901
		150	-0.0558	-0.0887	1.7504
		200	-0.0903	0.1133	1.5485
	0.6	50	-0.1879	-0.3121	0.8873
		100	0.2481	-0.1502	1.3189
		150	0.1969	0.1162	0.9772
		200	-0.2038	-0.3114	0.8311

Table S2. The relative improvement percentage of $SRC@k = 20$ in the WHEAT.CP dataset

γ	h^2	n_t	$MSPE_{(v2)}^{Ridge}$	$CD_{mean(v2)}$	$GV_{average}$
0.5	0.3	50	-7.8407	-4.1477	24.4413
		100	-6.3435	-7.4234	14.8341
		150	1.5006	7.3700	7.2948
		200	-3.0951	-5.0399	5.5086
	0.6	50	-1.0163	-2.0378	9.2073
		100	-2.4819	-2.1689	4.9679
		150	-3.0254	-0.4520	-4.6684
		200	-1.8233	-2.6989	-1.5932
1	0.3	50	0.0512	-4.2221	13.7099
		100	-10.5021	-8.5730	5.1485
		150	-3.9673	2.0476	3.5644
		200	-0.6309	5.3346	6.8007
	0.6	50	-7.4324	-0.5634	7.8126
		100	-7.4757	-9.6463	1.1815
		150	0.7835	4.2387	4.0604
		200	0.8009	-0.1507	5.2345
2	0.3	50	7.8460	11.0650	28.9342
		100	-0.7158	-7.8636	17.3342
		150	2.1556	7.0173	15.3886
		200	-2.7407	-4.5662	5.9788
	0.6	50	-3.0389	-9.3390	7.6598
		100	-3.8751	-1.3701	10.4100
		150	0.8770	8.8113	9.8367
		200	-2.3468	2.1734	7.6192
4	0.3	50	1.2290	-0.9823	14.6475
		100	-7.3106	-1.3877	16.5598
		150	2.8434	2.5678	10.7103
		200	-2.9198	-0.5605	12.9035
	0.6	50	3.4219	-0.4794	18.5932
		100	-8.9545	-9.1906	5.2614
		150	-0.2189	1.2640	1.5688
		200	0.9694	-3.0150	5.6855

Table S3. The relative improvement percentage of $RS_{-ratio}@k = 20$ in the WHEAT.CP dataset

γ	h^2	n_t	$MSPE_{(v2)}^{Ridge}$	$CD_{mean(v2)}$	$GV_{average}$
0.5	0.3	50	-2.1663	-2.5399	8.7770
		100	-0.3932	-0.5543	11.4327
		150	-0.5995	-0.4547	7.1749
		200	-2.8998	-2.2865	4.8445
	0.6	50	-1.5430	-1.5569	3.2420
		100	-1.4810	-2.3794	2.9219
		150	0.4742	-1.1477	0.3052
		200	-2.2014	-1.9186	0.3479
1	0.3	50	-2.1097	-0.9331	5.2560
		100	-1.0725	-1.1742	10.7213
		150	-1.8432	-1.0039	6.2480
		200	-1.9604	-2.7868	5.0230
	0.6	50	0.5500	-1.0415	6.1255
		100	-0.8684	-2.4822	5.2357
		150	0.0258	0.5409	4.1847
		200	-0.8134	-0.1826	4.9402
2	0.3	50	-1.5916	-3.0013	5.8698
		100	-0.1006	-2.9629	10.9033
		150	0.3414	-0.3961	9.0578
		200	-2.2525	-1.4492	7.1125
	0.6	50	0.4182	-1.6403	2.4069
		100	0.0605	-0.5228	8.6283
		150	0.4921	1.1384	7.4403
		200	-1.5721	0.1483	6.8021
4	0.3	50	-1.1173	-0.6690	6.6036
		100	0.5241	-2.8890	10.9101
		150	0.4317	0.3284	11.0724
		200	-2.0491	-0.3426	9.2106
	0.6	50	-2.7597	-1.8494	5.3301
		100	2.3958	-0.2162	11.7741
		150	1.4879	0.6710	9.4054
		200	-1.7648	-2.5801	7.8524

Table S4. The relative improvement percentage of $NDCG@k = 20$ in the MAIZE.CP dataset

γ	h^2	n_t	$MSPE_{(v2)}^{Ridge}$	$CD_{mean(v2)}$	$GV_{average}$
0.5	0.3	50	-1.3740	-1.7551	7.4887
		100	-1.7478	0.2814	5.8048
		150	-0.9460	0.7296	4.9199
		200	-0.3903	-0.5987	3.8752
	0.6	50	-1.2889	-1.6698	6.1610
		100	-1.1678	0.5147	4.6108
		150	-0.6860	0.6187	3.6888
		200	-0.3457	-0.5281	2.8552
1	0.3	50	-0.8491	-1.7194	7.4068
		100	-1.6343	0.2332	5.8238
		150	-0.4832	0.7588	4.9709
		200	-0.0666	-0.4435	3.9396
	0.6	50	-1.2752	-1.6392	5.8427
		100	-1.3640	0.3657	4.3362
		150	-0.6073	0.5364	3.6897
		200	-0.3168	-0.6805	2.9331
2	0.3	50	-1.4195	-2.0748	7.6027
		100	-1.6605	0.2654	6.4563
		150	-0.5719	0.7366	5.2011
		200	-0.0835	-0.4612	4.2537
	0.6	50	-0.9191	-1.5556	5.8314
		100	-1.0572	0.3251	4.7073
		150	-0.7278	0.3327	3.9231
		200	-0.2051	-0.3018	3.3503
4	0.3	50	-1.4190	-1.4902	6.9125
		100	-1.6780	0.2942	5.6244
		150	-0.8347	0.7461	4.7805
		200	-0.0478	-0.6126	4.0023
	0.6	50	-0.7329	-1.2849	5.5493
		100	-1.5232	0.4108	4.1575
		150	-0.3868	0.7640	3.9338
		200	-0.1076	-0.4842	3.2452

Table S5. The relative improvement percentage of $SRC@k = 20$ in the MAIZE.CP dataset

γ	h^2	n_t	$MSPE_{(v2)}^{Ridge}$	$CD_{mean(v2)}$	$GV_{average}$
0.5	0.3	50	-26.0798	-31.3725	236.7400
		100	-21.9485	13.6312	114.2160
		150	-14.8687	12.6031	83.1620
		200	-8.6591	-10.2804	59.6912
	0.6	50	-38.8868	-37.8096	171.1329
		100	-23.4348	14.8167	104.0615
		150	-11.9885	8.7859	70.1388
		200	-8.5353	-12.1335	47.9923
1	0.3	50	-29.7638	-46.9413	184.0376
		100	-25.7157	14.0888	126.1590
		150	-9.1720	17.6286	83.4963
		200	-9.1264	-13.8150	59.0705
	0.6	50	-26.2832	-48.0056	182.5251
		100	-28.0750	8.7870	92.7849
		150	-6.5984	14.2742	74.7213
		200	-9.5354	-16.5039	48.0615
2	0.3	50	-21.8801	-32.4254	214.2129
		100	-24.3427	5.6141	108.5019
		150	-2.7739	13.2779	88.7452
		200	-5.9092	-9.2418	65.5885
	0.6	50	-14.2172	-27.7389	250.5339
		100	-22.2901	9.1062	111.1978
		150	-12.5522	8.5386	79.2802
		200	-8.3520	-7.6542	62.8824
4	0.3	50	-28.6842	-43.0257	249.3312
		100	-33.5189	-2.0124	111.4493
		150	-5.5745	19.0306	91.8402
		200	-6.5405	-10.2351	63.7024
	0.6	50	-33.9032	-45.4344	163.2797
		100	-28.6632	6.5520	94.3671
		150	-5.1928	15.0356	79.5172
		200	-4.8847	-13.7742	58.5314

Table S6. The relative improvement percentage of $RS_{-ratio}@k = 20$ in the MAIZE.CP dataset

γ	h^2	n_t	$MSPE^{Ridge}_{(v2)}$	$CD_{mean(v2)}$	$GV_{average}$
0.5	0.3	50	-4.8408	-4.8604	33.3506
		100	-7.9412	-0.8046	34.9773
		150	-3.6965	3.7901	33.5908
		200	-0.1389	-1.4505	28.0612
	0.6	50	-5.1128	-4.7794	32.2503
		100	-6.1539	-0.2095	34.0737
		150	-3.2655	4.2275	31.0796
		200	-1.2305	-3.1025	25.0178
1	0.3	50	-2.5068	-3.6580	31.6346
		100	-6.2330	-0.2146	34.0974
		150	-2.8228	2.8503	30.8562
		200	0.0009	-1.8892	25.9282
	0.6	50	-4.9526	-5.2175	30.1345
		100	-7.4780	-0.0636	32.6016
		150	-5.0272	1.5245	29.6000
		200	-2.2292	-3.3388	24.8071
2	0.3	50	-5.8232	-5.6421	31.2316
		100	-8.1959	-2.2171	35.4074
		150	-2.9492	3.9842	34.1335
		200	-0.9198	-1.7286	27.5082
	0.6	50	-3.4023	-4.8850	28.6109
		100	-5.2910	0.5833	34.0774
		150	-5.8235	1.2399	30.7201
		200	-0.5347	-1.7149	28.9707
4	0.3	50	-6.7379	-4.4506	24.2639
		100	-5.8494	-0.6486	30.4411
		150	-3.8259	3.1394	29.8175
		200	0.4281	-1.6846	26.3922
	0.6	50	-2.0763	-1.8775	25.4709
		100	-9.3671	-1.2404	28.6171
		150	-3.9195	4.0871	31.0224
		200	-0.8951	-2.6815	27.2954

Table S7. The relative improvement percentage of $NDCG@k = 20$ in the RICE.CP dataset

γ	h^2	n_t	$\mathrm{MSPE}^{\mathrm{Ridge}}_{\mathrm{(v2)}}$	$\mathrm{CD}_{\mathrm{mean(v2)}}$	$\mathrm{GV}_{\mathrm{average}}$
0.5	0.3	50	-0.6459	0.3766	0.1980
		100	0.5203	0.2451	2.1025
		150	-0.0149	0.2936	2.0215
		200	0.1739	-0.0409	1.7536
	0.6	50	-0.4007	0.1518	-2.8690
		100	0.3415	0.0911	0.2930
		150	-0.2291	-0.0778	0.4114
		200	-0.0018	-0.0094	0.3484
1	0.3	50	-0.6897	0.8042	0.3911
		100	0.1107	-0.3026	1.5527
		150	-0.1098	-0.0556	1.6381
		200	0.1385	-0.2920	1.4661
	0.6	50	-0.2869	0.4607	-2.0311
		100	0.2253	-0.2022	0.4981
		150	-0.4623	-0.0133	0.5355
		200	0.0396	-0.0625	0.6857
2	0.3	50	-0.8614	1.1170	0.7294
		100	0.0433	-0.1019	1.6298
		150	-0.3237	0.4109	1.6459
		200	-0.1216	-0.4019	1.0399
	0.6	50	-0.4461	0.5245	-2.0572
		100	-0.0192	-0.3091	0.1940
		150	-0.2495	0.1504	0.5898
		200	-0.1149	-0.1945	0.6180
4	0.3	50	-0.6238	1.0381	-0.1325
		100	0.3454	-0.3459	1.4328
		150	-0.0403	0.1810	1.8417
		200	0.2363	-0.0037	1.3545
	0.6	50	-0.2724	0.5882	-1.8565
		100	0.0947	-0.5044	0.3712
		150	-0.5166	-0.2737	0.3468
		200	-0.0459	-0.1907	0.5583

Table S8. The relative improvement percentage of $SRC@k = 20$ in the RICE.CP dataset

γ	h^2	n_t	$MSPE^{Ridge}_{(v2)}$	$CD_{mean(v2)}$	$GV_{average}$
0.5	0.3	50	-21.1967	1.4346	37.5983
		100	8.5645	7.3167	43.9652
		150	-6.4324	-0.6557	28.2105
		200	-1.9828	4.5099	21.9036
	0.6	50	-9.8466	-2.4319	27.7662
		100	3.6419	6.5755	25.2344
		150	-6.6236	0.4992	17.9583
		200	-3.7660	1.9326	7.1251
1	0.3	50	-4.8124	8.5581	48.3661
		100	-5.1963	-1.9175	34.0089
		150	-13.4244	-4.4792	22.6361
		200	-1.3118	5.2621	21.0092
	0.6	50	-16.7389	-7.9747	26.4659
		100	-1.2903	-0.8468	23.1728
		150	-8.2395	-1.9529	13.2653
		200	-4.3838	-0.2594	9.9390
2	0.3	50	-16.6516	4.1176	54.9979
		100	-0.6020	3.4621	49.1965
		150	-3.7869	4.7623	36.4295
		200	-4.0782	-0.3326	22.4690
	0.6	50	-7.5426	8.0842	39.9720
		100	-4.7580	-1.8053	21.1852
		150	-8.0397	-1.6902	18.8728
		200	-2.2971	1.4516	12.0989
4	0.3	50	-11.3652	1.9168	55.9266
		100	-0.1140	0.1252	36.8332
		150	-12.1308	-2.3682	30.3424
		200	-1.4975	1.6439	20.0267
	0.6	50	-12.7049	1.9269	35.2756
		100	-5.3271	-5.9431	29.6335
		150	-11.1465	-5.5473	17.1908
		200	-2.9159	0.2904	12.8654

Table S9. The relative improvement percentage of $RS_{\_ratio}@k = 20$ in the RICE.CP dataset

γ	h^2	n_t	$MSPE^{Ridge}_{(v2)}$	$CD_{mean(v2)}$	$GV_{average}$
0.5	0.3	50	-1.7416	2.0267	0.0054
		100	3.5554	1.0434	8.5059
		150	1.0526	1.5585	9.3127
		200	1.4658	-1.2327	9.2452
	0.6	50	-1.1042	3.0791	-15.3378
		100	3.0397	0.0828	0.2763
		150	-2.9108	-0.6613	1.0676
		200	0.2715	-0.9647	1.4779
1	0.3	50	-3.8816	4.1835	0.2835
		100	1.4648	-2.3664	6.5904
		150	-1.3653	-0.8591	5.9227
		200	0.2218	-2.9821	7.9246
	0.6	50	-1.1205	5.1147	-12.0600
		100	1.8428	-1.5052	0.7603
		150	-3.4740	-0.9664	1.2502
		200	0.6228	-0.2045	3.9146
2	0.3	50	-1.6248	7.3742	0.9294
		100	0.7837	-2.1061	4.5342
		150	-2.4067	1.8655	6.6853
		200	0.6437	-2.6667	5.2265
	0.6	50	-2.8245	2.8243	-12.3079
		100	-0.0007	-1.7750	-1.0702
		150	-2.2131	0.3363	2.4731
		200	-0.6318	-1.3419	4.1267
4	0.3	50	-1.9967	5.4366	-3.6485
		100	2.4271	-1.8617	4.4934
		150	0.0304	1.1722	6.5918
		200	0.8869	0.0097	5.7826
	0.6	50	-0.1093	3.6028	-11.3811
		100	1.5334	-2.6284	0.7290
		150	-2.5712	-1.6462	1.7851
		200	0.1952	-0.7819	4.7210

Table S10. The $\mathrm{MSPE}_{(v2)}^{\mathrm{Ridge}}$ values and rankings (in parentheses) for the 20 random training sets sampled from the MAIZE.CP dataset under the different parameter settings. The data of S1−S20 are available online (https://github.com/spcspin/AD).

	λ				
Sample	0.01	0.1	1	10	100
S1	(1) 2890593	(1) 2890593	(1) 2890593	(1) 2890609	(1) 2892119
S2	(2) 2868948	(2) 2868948	(2) 2868948	(2) 2868954	(2) 2869608
S3	(3) 2866996	(3) 2866996	(3) 2866996	(3) 2867004	(3) 2867861
S4	(4) 2866019	(4) 2866019	(4) 2866019	(4) 2866027	(4) 2866830
S5	(5) 2865514	(5) 2865514	(5) 2865514	(5) 2865519	(5) 2866073
S6	(6) 2862410	(6) 2862410	(6) 2862410	(6) 2862421	(6) 2863553
S7	(7) 2828472	(7) 2828472	(7) 2828472	(7) 2828483	(7) 2829562
S8	(8) 2792809	(8) 2792809	(8) 2792809	(8) 2792815	(8) 2793448
S9	(9) 2785898	(9) 2785898	(9) 2785898	(9) 2785911	(9) 2787213
S10	(10) 2783844	(10) 2783844	(10) 2783844	(10) 2783851	(10) 2784590
S11	(11) 2778484	(11) 2778484	(11) 2778484	(11) 2778493	(11) 2779435
S12	(12) 2768766	(12) 2768766	(12) 2768766	(12) 2768772	(12) 2769405
S13	(13) 2768374	(13) 2768374	(13) 2768374	(13) 2768381	(13) 2769179
S14	(14) 2763846	(14) 2763846	(14) 2763845	(14) 2763852	(14) 2764589
S15	(15) 2760086	(15) 2760086	(15) 2760086	(15) 2760091	(15) 2760654
S16	(16) 2746613	(16) 2746613	(16) 2746612	(16) 2746617	(16) 2747143
S17	(17) 2742414	(17) 2742414	(17) 2742414	(17) 2742421	(17) 2743249
S18	(18) 2722849	(18) 2722849	(18) 2722849	(18) 2722858	(18) 2723790
S19	(19) 2718772	(19) 2718772	(19) 2718772	(19) 2718784	(19) 2720002
S20	(20) 2707398	(20) 2707398	(20) 2707398	(20) 2707404	(20) 2708074

Table S11. The $\mathrm{CD}_{\mathrm{mean(v2)}}$ values and rankings (in parentheses) for the 20 random training sets sampled from the MAIZE.CP dataset under the different parameter settings. The data of S1−S20 are available online (https://github.com/spcspin/AD).

	(σ_A^2, σ_D^2)				
Sample	(0.5,0.5)	(0.5,1)	(1,1)	(1,2)	(1,4)
S1	(1) 148.9192	(1) 162.4199	(1) 183.3370	(1) 191.8792	(1) 201.8588
S2	(2) 148.7217	(2) 162.1164	(2) 182.8530	(2) 191.4933	(2) 201.6517
S3	(4) 146.9321	(4) 160.4259	(3) 181.5909	(3) 190.2929	(3) 200.5342
S4	(3) 147.2447	(3) 160.5621	(4) 180.8421	(4) 189.4448	(6) 199.5636
S5	(5) 146.4483	(5) 159.8770	(5) 180.6693	(5) 189.4111	(4) 199.7492
S6	(6) 146.2759	(6) 159.1419	(6) 179.8157	(9) 187.8775	(10) 197.4507
S7	(8) 145.0924	(8) 159.0085	(7) 179.6957	(6) 188.8411	(7) 199.4259
S8	(7) 145.6667	(7) 159.0819	(8) 179.4245	(8) 188.0635	(9) 198.0748
S9	(10) 143.6522	(9) 158.0280	(9) 178.9141	(7) 188.5685	(5) 199.7348
S10	(9) 143.9703	(10) 157.9481	(10) 178.4500	(10) 187.7346	(8) 198.4845
S11	(11) 143.3669	(11) 157.0991	(11) 177.9608	(11) 186.8308	(11) 197.1074
S12	(14) 141.3257	(13) 154.9387	(12) 175.4978	(12) 184.4108	(12) 194.7359
S13	(12) 143.3053	(12) 155.5582	(13) 175.2466	(14) 182.7115	(15) 191.4924
S14	(13) 141.5702	(14) 154.8961	(14) 174.4476	(13) 183.3728	(13) 193.9011
S15	(16) 139.2144	(15) 152.6814	(15) 172.0465	(15) 181.1853	(14) 191.8513
S16	(15) 139.4605	(18) 151.7012	(16) 171.8677	(18) 179.5527	(20) 188.7909
S17	(18) 138.5266	(16) 152.0760	(17) 171.5355	(16) 180.4924	(16) 190.8543
S18	(17) 139.1739	(17) 151.9577	(18) 171.3846	(17) 179.7363	(18) 189.5318
S19	(19) 137.4673	(19) 150.4835	(19) 169.8855	(20) 178.6038	(19) 188.8517
S20	(20) 135.0686	(20) 149.2592	(20) 169.4391	(19) 178.9876	(17) 189.8759
